# Detection and characterization of mineralo-organic nanoparticles in human kidneys

**DOI:** 10.1038/srep15272

**Published:** 2015-10-26

**Authors:** Tsui-Yin Wong, Cheng-Yeu Wu, Jan Martel, Cheng-Wei Lin, Fu-Yung Hsu, David M. Ojcius, Paul Y. Lin, John D. Young

**Affiliations:** 1 Laboratory of Nanomaterials, Chang Gung University, Gueishan, Taoyuan, Taiwan, Republic of China; 2 Center for Molecular and Clinical Immunology, Chang Gung University, Gueishan, Taoyuan, Taiwan, Republic of China; 3 Research Center of Bacterial Pathogenesis, Chang Gung University, Gueishan, Taoyuan, Taiwan, Republic of China; 4 Department of Materials Engineering, Ming Chi University of Technology, Taishan, New Taipei City, Taiwan, Republic of China; 5 Department of Biomedical Sciences, University of the Pacific, Arthur Dugoni School of Dentistry, San Francisco, California, USA; 6 Department of Pathology, Chia-Yi Chang Gung Memorial Hospital, Puzi, Chia-Yi, Taiwan, Republic of China; 7 Laboratory of Cellular Physiology and Immunology, Rockefeller University, New York, NY, USA; 8 Biochemical Engineering Research Center, Ming Chi University of Technology, Taishan, New Taipei City, Taiwan, Republic of China

## Abstract

Ectopic calcification is associated with various human diseases, including atherosclerosis, cancer, chronic kidney disease, and diabetes mellitus. Although mineral nanoparticles have been detected in calcified blood vessels, the nature and role of these particles in the human body remain unclear. Here we show for the first time that human kidney tissues obtained from end-stage chronic kidney disease or renal cancer patients contain round, multilamellar mineral particles of 50 to 1,500 nm, whereas no particles are observed in healthy controls. The mineral particles are found mainly in the extracellular matrix surrounding the convoluted tubules, collecting ducts and loops of Henle as well as within the cytoplasm of tubule-delineating cells, and consist of polycrystalline calcium phosphate similar to the mineral found in bones and ectopic calcifications. The kidney mineral nanoparticles contain several serum proteins that inhibit ectopic calcification in body fluids, including albumin, fetuin-A, and apolipoprotein A1. Since the mineralo-organic nanoparticles are found not only within calcified deposits but also in areas devoid of microscopic calcifications, our observations indicate that the nanoparticles may represent precursors of calcification and renal stones in humans.

Ectopic calcification is associated with atherosclerosis, cancer, chronic kidney disease, and diabetes mellitus^1^,^2^,^3^. Recent studies indicate that atherosclerosis and chronic kidney disease patients with signs of vascular calcification show increased morbidity and mortality risks, suggesting that ectopic calcification is detrimental to human health^1^,^3^. Unwanted calcification is also observed in aging individuals, and most people over the age of 60 show signs of vascular calcification^4^. For these reasons, deciphering the factors that induce ectopic calcification and developing effective treatment represent important goals.

Ectopic calcification can be defined as an imbalance between inhibitors and inducers of calcification in the body. Calcification inhibitors include serum proteins such as albumin, fetuin-A, osteopontin, and matrix gla protein as well as small compounds like pyrophosphate, whereas hyperphosphatemia and inflammation represent major inducers of calcification^5^,^6^,^7^. Recent studies indicate that calcification inducers activate a cellular process similar to bone formation during vascular calcification^7^,^8^. Matrix vesicles similar to the ones that induce mineralization in developing bones have also been detected in calcified soft tissues^7^,^8^,^9^. These matrix vesicles are probably released by vascular smooth muscle cells that differentiate into osteoblast-like cells, which induce calcification^7^.

Mineral nanoparticles (NPs) have been detected in soft tissues showing signs of ectopic calcification. Price *et al.* found that the serum of rats treated with either the bisphosphonate etidronate or with vitamin D harbor mineralo-protein complexes containing the calcification inhibitors fetuin-A and matrix gla protein^10^,^11^. Similarly, Jahnen-Dechent *et al.* observed that fetuin-A-containing mineral complexes, which were called calciprotein particles (CPPs), could be detected in the ascitic fluid of patients with calcifying peritonitis^12^. A recent study by Bertazzo *et al.* demonstrated the presence of mineral NPs in the aortic valves and coronary arteries of both atherosclerotic and rheumatic fever patients^13^. While studies on the formation of mineral particles have usually focused on the human cardiovascular system, it remains unclear whether the particles may be found in other tissues and whether these entities play a role in health or disease.

We observed earlier that mineralo-organic NPs spontaneously form in human and animal body fluids^14^,^15^,^16^,^17^,^18^,^19^,^20^,^21^,^22^,^23^,^24^,^25^,^26^,^27^,^28^. These mineral NPs were initially described as nanobacteria (NB) and were believed to be not only the smallest cells on earth^29^, but also a possible transmissible cause of numerous diseases, including Alzheimer’s disease, atherosclerosis, cancer, kidney stone formation, polycystic kidney disease, and prostatitis^30^,^21^,^32^. However, our results have shown that NB are in fact non-living mineral NPs that mimic common bacteria in terms of their morphology, growth, proliferation, and sub-culture^15^,^18^,^22^. The possibility that mineral particles similar to the so-called NB may be found in human tissues and whether these particles play a role in disease remains to be examined.

In the present study, we developed a nanomaterial approach to detect and analyze mineralo-organic NPs in diseased human kidney tissues. We show that kidney tissues from end-stage chronic kidney disease and renal cancer patients contain multilamellar mineral NPs similar to the biomimetic mineral particles that spontaneously precipitate in body fluids *in vitro*. Our results reveal critical insights concerning the biochemical composition, mechanism of formation and biological function of these mineralo-organic particles and they shed light on mechanisms of ectopic calcification and initiation of disease in the human body.

## Results

We examined kidney tissues surgically removed from human patients with either end-stage chronic kidney disease (n = 2) or renal cancer (n = 18; see Table 1; for renal cancer samples, we focused on the non-cancerous part of the tissue). As healthy controls, we studied kidney biopsies obtained from patients with either trauma or hematoma but who had no prior abnormal renal function (n = 2; Table 1).

**Figure 1:**
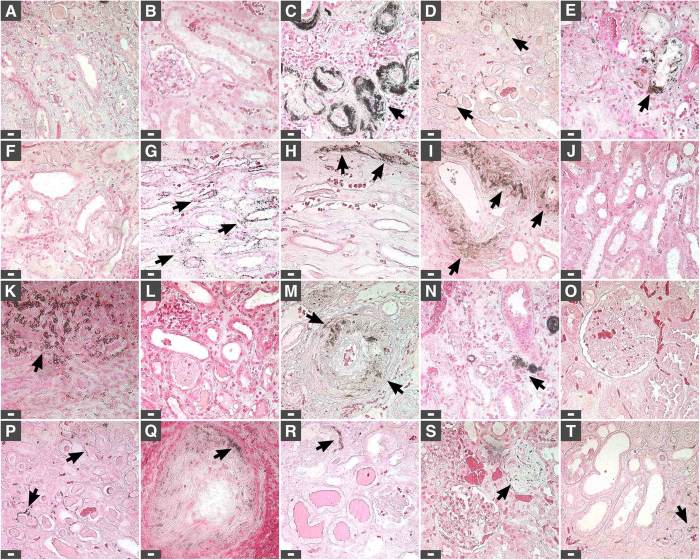
von Kossa staining of human kidney tissues. Kidney tissues were processed for von Kossa staining as described in *Methods*. Positive staining was based on the observation of black precipitates under optical microscopy (black arrows). Tissues in (**A**,**B**) consist of healthy controls; (**C**,**D**) correspond to patients with end-stage kidney disease; (**E**–**T**) tissues from renal cancer patients. Tissues from two additional cases of renal cancer are shown in Fig. 2. Scale bars: 10 μm.

**Figure 2:**
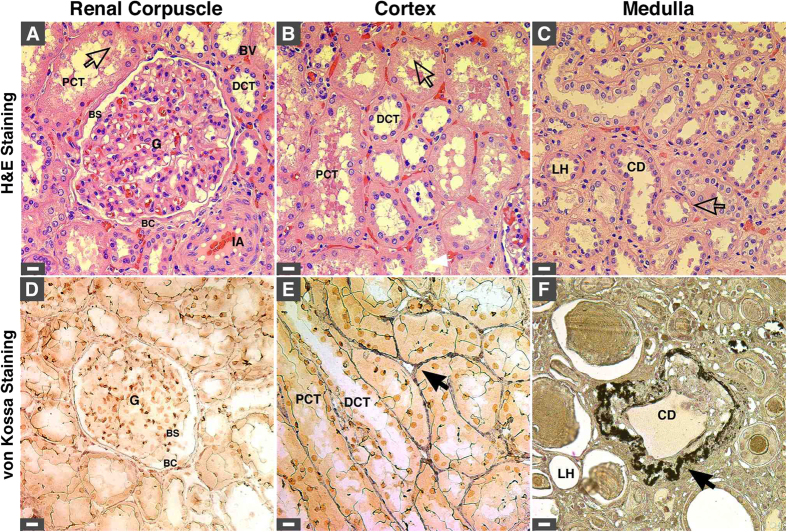
Mineral deposits found in diseased human kidneys. (**A**–**C**) H&E staining of human kidney tissues obtained from renal cancer patients. Hollow arrows indicate areas with cellular damage. (**D**–**F**) von Kossa staining of human kidney tissues. Mineral precipitates indicated by black arrows were found in the cortex (**E**) and medulla (**F**) but were absent in the renal corpuscle (**D**). Ectopic mineralization was found mainly in the basal membrane and interstitium surrounding the PCT, DCT, LH, and CD. Abbreviations: BC, Bowman’s capsule; BS, Bowman’s space; BV, blood vessel; CD, collecting duct; DCT, distal convoluted tubule; G, glomerulus; IA, interlobular artery; LH, loop of Henle; PCT, proximal collecting tubule. Scale bars: 10 μm.

Kidney tissues from healthy individuals showed normal histological features, and no ectopic calcification was observed following von Kossa staining (Fig. 1A,B). On the other hand, kidney tissues obtained from diseased individuals and stained with hematoxylin and eosin (H&E) showed evidence of tissue damage (Fig. 2A–C, indicated by arrows), and 80% of the diseased tissues examined showed mineralized deposits as revealed by von Kossa staining (Fig. 1C–T, Fig. 2E,F, calcification is visible as black material indicated by black arrows; see also Table 1). Calcified deposits were noticed in the cortex and medulla, including the extracellular space surrounding the distal convoluted tubules, proximal convoluted tubules, collecting ducts and loops of Henle as well as the cytoplasm of cells delineating the tubules and ducts (Fig. 1C–T and Fig. 2E,F). No calcification was detected in the renal corpuscle (Fig. 2D).

**Figure 3:**
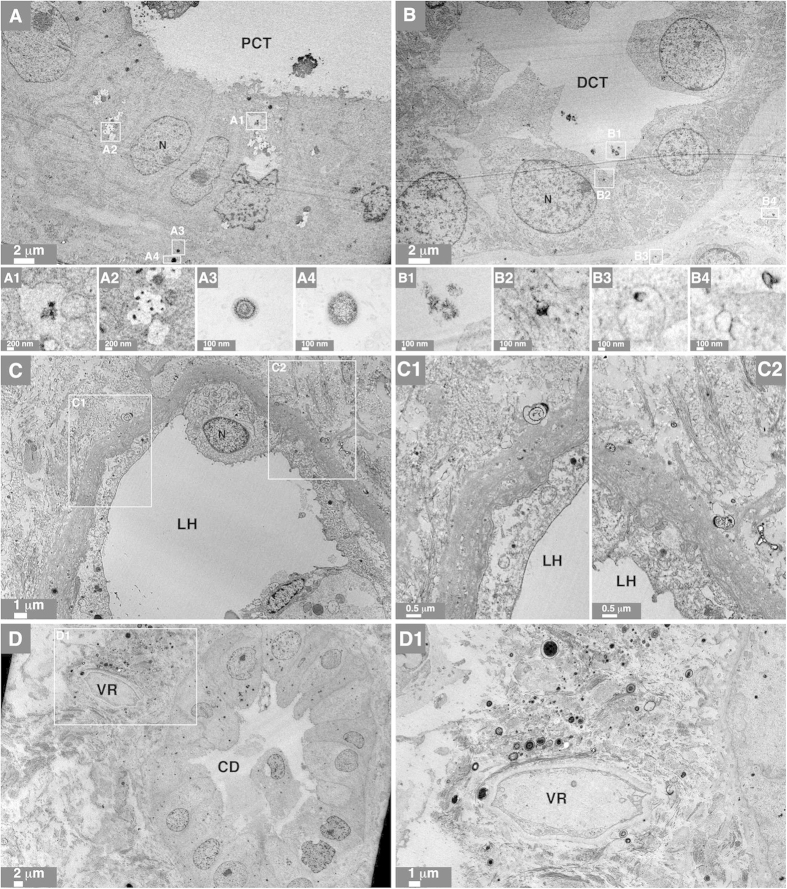
Ultrastructural localization of mineral NPs in diseased human kidney tissues. (**A**–**D**) Human kidney tissues were prepared for thin-section TEM as described in *Methods*. Mineral NPs were observed in the cytoplasm of epithelial cells as well as in the extracellular matrix and lumen of (**A**) PCT, (**B**) DCT, (**C**) LH, and (**D**) CD. Images A1-A4, B1-B4, C1, C2, and D1 correspond to enlarged sections denoted by white rectangles in panels (**A**–**D**). Note that the particles in A1 and A2 are surrounded by a cellular membrane. The VR in panel D1 is surrounded by a large number of NPs. Abbreviations: N, nucleus; VR, vasa recta renis.

To examine the nature of the mineral precipitates, we prepared ultra-thin kidney sections for observation by transmission electron microscopy (TEM). In specimens containing microscopic mineral deposits, mineral particles or granules were detected in the cytoplasm of renal epithelial cells, in the extracellular matrix beneath the basement membrane, and within the lumen of proximal and distal convoluted tubules (Fig. 3A,B, particles are enlarged in panels A1–A4 and B1–B4). Mineral NPs were also observed within cells lining the loops of Henle and collecting ducts (Fig. 3C,D, enlarged in panels C1, C2, and D1). Some particles were found within intracellular vesicles in renal cells (Fig. 3A, panels A1 and A2). The electron microscopy approach used here also allowed us to visualize the interior of the mineral particles; some particles harbored electron-dense rings alternating with light, electron-lucent layers (Fig. 3A, panels A3 and A4, Fig. 3C, panel C1). Several blood vessels, such as the vasa recta renis (the straight arteries of the kidney), were surrounded by large numbers of mineral NPs (Fig. 3D, panel D1). Mineral NPs were thus found at various locations in all human kidney tissues showing signs of ectopic calcification, whereas no particles were found in the healthy controls examined.

**Figure 4:**
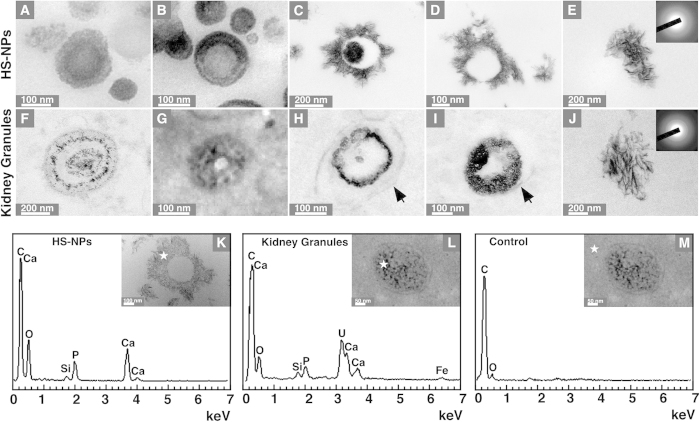
Morphology and chemical composition of mineralo-organic NPs and mineral particles found in kidney tissues. (**A**–**E**) Mineralo-organic NPs labeled as HS-NPs were prepared by adding 3 mM CaCl_2_ and Na_2_HPO_4_ each into DMEM containing 10% HS, followed by incubation for one week in cell culture conditions. TEM observations of HS-NP thin sections showed round particles with a smooth or crystalline surface. (**F**–**J**) Mineral particles or granules observed in human renal tissues by thin-section TEM. Kidney NPs or granules showed morphologies and sizes similar to that of HS-NPs. Some kidney granules were surrounded by a lipid membrane ((**H**,**I**) black arrows) while membranes were not observed in HS-NP specimens prepared *in vitro*. Representative electron diffraction patterns are shown in (**E**) and (**J**) (insets). EDX analysis of (**K**) prepared HS-NPs, (**L**) kidney granules found in human renal tissues, and (**M**) control kidney tissues. White stars indicate the areas selected for EDX analysis. Both HS-NPs and kidney granules show major peaks of carbon, calcium, oxygen and phosphorus along with other minor peaks, consistent with the presence of calcium phosphate containing additional ions. (**M**) EDX spectrum of control tissues surrounding the particles show no calcium or phosphate peaks. Ca:P ratios: (**K**) 1.18; (**L**) 0.65.

The mineral particles found in kidney tissues appear to be very similar to the mineralo-organic NPs described to form spontaneously in body fluids in our previous studies^15^,^17^ (and which we have termed *bions*^24^). To verify this possibility, we prepared mineralo-organic NPs (or *bions*) using a precipitation method as we previously described^15^. This method consists of adding precipitating ions like calcium and phosphate into a cell culture medium (Dulbecco’s modified Eagle’s medium or DMEM) containing a body fluid like human serum (HS), followed by incubation in cell culture conditions (see *Methods*). The particles produced this way (HS-NPs) were either spherical or ellipsoid and showed a smooth or crystalline mineral surface (Fig. 4A–E), similar to the particles described earlier in body fluids^22^,^23^ as well as in human ascites^12^ and calcified arteries^13^. The mineral particles were very similar to the mineral NPs or granules observed in kidney tissues in terms of their overall morphology, multilayer structure, and surface characteristics (Fig. 4F–J). The size of HS-derived particles and kidney granules were also comparable, varying from 50 to 1,500 nm in diameter (Fig. 4A–E,F–J). As noted above, some kidney granules were surrounded by a lipid membrane, possibly representing intracellular cargo vesicles or extracellular membrane vesicles (Fig. 4H,I, membranes are denoted by arrows); such membranous structures were absent in HS-NP specimens prepared *in vitro* (Fig. 4A–E). These observations suggest that kidney granules are similar to the mineralo-organic NPs assembled in serum.

Using selected area electron diffraction analysis, we observed that the mineral phase of HS-NPs prepared *in vitro* consisted of a polycrystalline nanomaterial (Fig. 4E, inset; notice the faint concentric rings). Similar results were obtained for kidney granules (Fig. 4J, inset) and for bones and ectopic calcifications as described in previous studies^33^,^35^.

We studied the chemical composition of HS-NPs and kidney granules using energy-dispersive X-ray spectroscopy (EDX). HS-NPs showed major peaks of carbon (C), calcium (Ca), oxygen (O), and phosphorus (P) (Fig. 4K), consistent with the presence of a calcium phosphate mineral. A low peak of silicon (Si) was also noted in HS-NPs (Fig. 4K), possibly representing a minor particle constituent. Kidney granules also showed peaks of carbon, calcium, oxygen and phosphorus, indicative of a calcium phosphate mineral, along with additional peaks of silicon and iron (Fe) (Fig. 4L). Uranium peaks (U) were attributed to the uranyl acetate used as contrasting reagent during sample preparation (the presence of uranium in some samples of mineral particles like kidney granules and its absence in the control tissue in Fig. 4M may be attributed to the high affinity of uranium for phosphate, as reported previously^35^). Control EDX spectra of kidney tissue surrounding the particles showed peaks of carbon and oxygen (Fig. 4M), suggesting that calcium and phosphorus were found mainly in the mineral particles.

Calcium:phosphorus (Ca:P) ratios of HS-NPs and kidney granules varied from 0.65 to 1.18. These ratios differ from the theoretical value of 1.67 observed for stoichiometric hydroxyapatite but are still within the range seen earlier for calcium phosphate and apatite crystals observed at various degrees of crystallization^15^. Together, these findings confirm that the kidney granules consist of calcium phosphate NPs.

Various proteins have been found to inhibit ectopic calcification in a systemic manner in the body^36^,^37^. In addition, the *protein corona* found on the surface of synthetic NPs is believed to determine the biodistribution and effects of the particles on cells *in vivo*^38^,^39^. On the other hand, the protein composition of mineralo-organic NPs found in human kidney tissues remains incompletely understood. We found previously that albumin, fetuin-A and apolipoprotein-A1 (apo-A1) represent the main proteins that interact with mineralo-organic NPs formed in body fluids^17^,^20^. Here we used immunogold labeling to examine the presence and ultrastructural location of these proteins within kidney granules.

**Figure 5:**
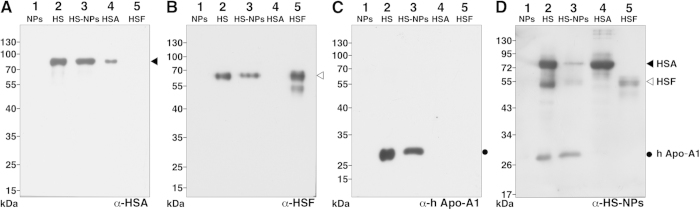
Specificity of polyclonal antibodies against serum proteins. (**A**–**D**) Polyclonal antibodies were prepared in rabbits as described earlier^25^. Western blotting was performed in reducing and denaturing conditions as described in *Methods*. A control specimen containing only mineral NPs was used in lane 1. Reactions against HSA, HSF, and human apo-A1 are indicated with a black triangle, a white triangle, or a black circle, respectively. (**A**) The anti-HSA antibody (α-HSA) reacted with a single band of 85 kDa in whole HS, HS-NPs and purified HSA but produced no reaction against a negative control of mineral NPs or HSF. The anti-HSF antibody (α-HSF) reacted with a 62-kDa band in whole HS and HS-NPs and with two bands of 55 and 62 kDa in HSF ((**B**) the presence of multiples bands for purified HSF was attributed to glycosylation which may affect protein migration). (**B**) No reaction was noted for the anti-HSF antibody against the negative NP control or HSA. (**C**) The anti-apo-A1 antibody (α-h Apo-A1) reacted with a 30-kDa band in whole HS and HS-NPs but produced no reaction against mineral NPs, HSA, or HSF. (**D**) The polyclonal anti-HS antibodies reacted with three bands of 30, 58, and 75 kDa in whole HS and HS-NPs (these protein bands likely corresponded to HSA, HSF, and apo-A1, respectively). In addition, the anti-HS antibody mainly produced a 75-kDa band against HSA and a 55-kDa band in the lane containing HSF. Given that purified proteins were used and that the molecular weights observed for HSA, HSF, and apo-A1 correspond to the molecular weight observed for these proteins in previous studies^15^,^16^ (approximately 28, 55, and 72 kDa, respectively), these results suggest that the polyclonal antibodies reacted positively and specifically against the serum proteins used as antigens.

**Figure 6:**
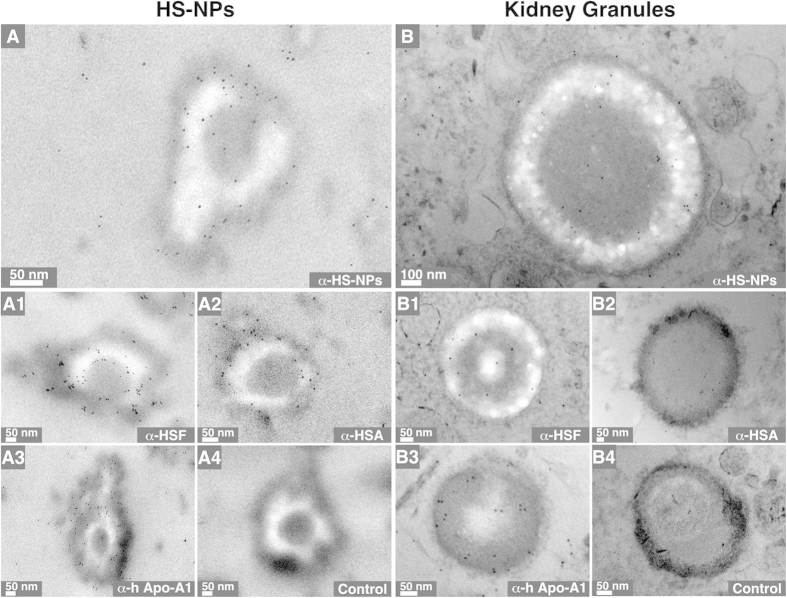
Immunogold staining of mineralo-organic NPs and kidney granules. (**A**) HS-NPs prepared as in Fig. 4 and (**B**) human kidney tissues were processed for thin-section TEM and immunogold labeling. The antibodies used are indicated at the bottom right corner of each panel. Treatment without primary antibody was used as a negative control (panels A4 and B4).

We used polyclonal antibodies against human serum albumin (HSA), human serum fetuin-A (HSF), human apo-1A, and whole HS to examine the presence of serum proteins in HS-NPs and kidney granules. The specificity of the polyclonal antibodies (prepared as described earlier^25^) was verified using Western blotting (Fig. 5). Each one of the polyclonal antibodies reacted positively with the HS-NPs prepared *in vitro* as well as with the mineral granules found in human kidney tissues (Fig. 6A,B, panels A1–A3 and B1–B3; black dots). The antibodies reacted mainly with the electron-dense layers or the dark core of HS-NPs and kidney granules (Fig. 6A,B), indicating that these dark areas may contain higher levels of proteins compared with electron-lucent areas. Negative controls performed without primary antibody produced no reaction (Fig. 6A,B, panels A4 and B4, control). We concluded that kidney granules represent mineralo-organic NPs similar to HS-NPs based not only on their morphology and mineral composition but also on their binding to the major calcification inhibitors present in serum.

**Figure 7:**
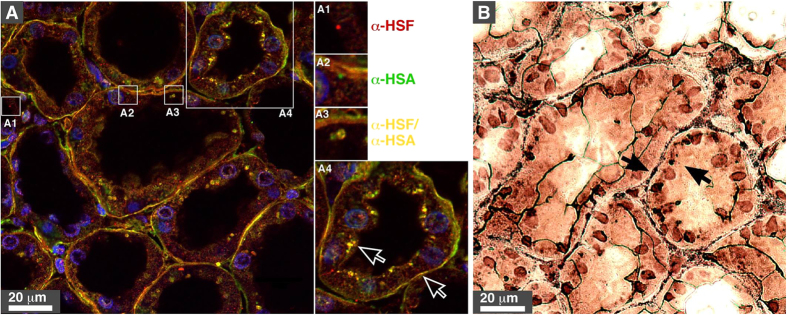
Immunofluorescence and von Kossa staining of human kidney tissues. (**A**) Polyclonal antibodies whose specificity was evaluated in Fig. 5A,B were used to stain renal tissues. Primary antibodies against HSA and HSF reacted with a secondary antibody labeled respectively with a red or green fluorophore in panels A1 and A2. Merged staining is shown in yellow (panels A, A3, and A4). Cell nuclei were stained with DAPI (blue). The images showed positively-stained tubules. Dots of proteineous particles were found in epithelial cells, cell cytoplasm, nuclei, and brush-like structures close to the lumen. (**B**) kidney tissues were stained with von Kossa. The pattern of ectopic calcification observed in (**B**) appeared to overlap to some extent with the merged yellow staining detected in (**A**), suggesting the presence of mineralo-organic particles in the cell cytoplasm and interstitium (hollow and black arrows).

Immunofluorescence microscopy was used next to confirm the presence of mineral granules containing HSA and HSF in diseased human kidneys. Using this technique, human kidney tissues showed positive staining for the two proteins in various areas, including the interstitium surrounding renal tubules as well as the cytoplasm of tubule-delineating cells (Fig. 7A, panels A1 and A2). Protein aggregates containing both albumin and fetuin-A were also noticed in these areas, albeit in lower amounts compared to the staining of the single proteins (Fig. 7A and panel A3, merged staining in yellow). Notably, we noticed that the protein staining detected by immunofluorescence closely overlapped with the pattern of ectopic calcification observed using von Kossa staining (Fig. 7A,B, calcification is visible as black material indicated by arrows in B). These results provide further support for the presence of mineralo-organic particles in the human kidney tissues examined.

## Discussion

While advances have been made in our understanding of the interactions between synthetic NPs and human cells, we know considerably less about the effects of mineralo-organic NPs that form spontaneously in body fluids. Our previous studies have shown that these particles form in biological fluids when the concentrations of calcium and phosphate exceed saturation^15^,^16^. We also observed that these particles are internalized by immune cells but that only large particles induce pro-inflammatory immune reactions^23^. However, the distribution of these particles in human tissues and whether they play any physiological or pathological roles in the body had not been examined so far.

In the present study, we detected for the first time mineralo-organic NPs in kidneys of human patients suffering from either end-stage kidney disease or renal cancer. The mineralo-organic NPs detected contain poorly crystallized calcium phosphate similar to bone mineral, as well as albumin, fetuin-A and apo-A1 which act as systemic calcification inhibitors in body fluids. Our results are in agreement with previous reports that described the presence of mineral-protein complexes in vascular tissues and body fluids^12^,^13^,^34^,^40^. Since the particles that we observed were found in areas containing no microscopic calcifications, our observations suggest that the particles may represent precursors of ectopic calcification in human tissues. Given the possibility that mineralo-organic NPs may gradually grow in size and undergo a particle-to-film conversion under favorable conditions as described in our previous studies^15^,^18^, the observations presented here suggest that the mineral precursors may lead to the formation of larger mineral deposits *in vivo*, such as Randall’s plaque and kidney stones.

Our observations that mineral ectopic calcifications and mineralo-organic NPs are found in various anatomical structures of the kidneys are consistent with previous findings of ectopic calcifications in this organ^41^. Evan *et al.*’s observations indicated that mineralization in the kidneys of patients with nephrolithiasis may start and occur predominantly in the interstitial tissue of the loops of Henle^40^,^42^. These authors observed that mineral deposits forming in this area may protrude from the basal side of the urothelium and lead to the formation of kidney stones. Our observations suggest that, in addition to the loops of Henle, other kidney areas may contain mineral NPs which may eventually evolve to form large mineral deposits in human kidneys. We are also investigating the possibility that mineral NPs that form in the blood circulation may translocate into renal tissues and induce ectopic calcification and stone formation in kidneys.

Several kidney granules detected in the present study are found within intracellular or extracellular vesicles (Fig. 4H,I) and these are similar to the matrix vesicles shown to induce calcification in bones and teeth^7^,^8^. We recently observed that vesicles isolated from human and animal serum induce the formation of mineral NPs and microscopic precipitates *in vitro*^25^. Schlieper *et al.*^34^ also observed that mineral particles found in arteries are associated with membrane structures and proposed that matrix vesicles or apoptotic bodies may represent nucleators of mineral particles in these tissues. Similarly, Khan *et al.* reported that calcium phosphate deposits found in the kidneys of human patients with idiopathic kidney stones are associated with collagen fibers and matrix vesicles^9^. These results suggest that the mineralo-organic NPs detected in kidney tissues may form via a mechanism involving membrane vesicles, a situation analogous to what is seen in atherosclerotic arteries^7^,^8^. On the other hand, a recent study showed that ectopic calcification found in women breast arteries was not associated with osteogenic or apoptotic cell markers^43^, suggesting that the mechanism of calcification may be specific to the organ or biological context involved. In addition, mineralo-organic NPs such as the ones described here in human kidney tissues may also form due to a failure to maintain physiological concentrations of ions (e.g., calcium and phosphate) and calcification inhibitors (e.g., albumin, fetuin-A, and apo-A1) in human body fluids.

Our results suggest that the electron-dense layer and core of mineralo-organic NPs may contain higher levels of proteins and organic molecules compared to the electron-lucent areas of the particles (Fig. 6A,B). These electron-dense layers appear to be mineralized as seen by the crystalline nature of this material under TEM (see Fig. 4A–J). Other authors, including Ryall^41^ and Evan *et al.*^44^, have proposed that the electron-lucent layer may represent minerals while electron-dense layers may correspond to organic molecules. These interpretations may be due at least in part to the manner in which the samples were processed and examined in each study as well as the nature of the starting tissues used.

In addition to playing a role in ectopic calcification, mineralo-organic particles may induce inflammation in kidney tissues. We have shown recently that while mineralo-organic NPs fail to induce the secretion of pro-inflammatory interleukin-1β by human macrophages, mineral aggregates larger than 1 μm are able to do so^23^. The release of interleukin-1β in response to crystalline materials has been shown to rely on the activation of intracellular molecular complexes termed the inflammasomes^45^,^46^,^47^. In addition, the mineral particles were detected in kidneys harboring tumors and the association between cancer and inflammation is now well recognized^48^. The possibility that aggregated mineral particles may activate an inflammasome and contribute to the development of inflammation and cancer in kidneys or other tissues remains to be investigated.

In addition to ectopic calcification, the mineral granules described here may participate in other disease processes. For instance, we have proposed earlier that mineral NPs may bind to various proteins in body fluids and deplete these organic molecules from body fluids^20^,^26^. On the other hand, the human body may prevent the formation and accumulation of mineral NPs under normal circumstances by relying on the presence of calcification inhibitors and the reticuloendothelium system (i.e., macrophages). Mineral NPs may thus accumulate only once systemic or local calcium homeostasis is perturbed and when the protective mechanisms have failed in the human body.

We propose that the nanomaterial approach developed here may be used to study the formation of mineralo-organic NPs in animal and human tissues. For instance, antibodies raised against calcification inhibitor proteins such as albumin, fetuin-A and apo-A1 may be used in combination with mineral analysis to detect and characterize the formation of mineralo-organic NPs in the tissues of animal models. The results obtained using this approach may be applied to clinical measurements made on human body fluids in order to identify biological parameters that reflect the state of mineral particle formation and ectopic calcification in the body. We expect that this knowledge may lead to the development of novel therapeutic strategies to prevent and treat human diseases and conditions associated with ectopic calcification.

## Methods

### Kidney tissues

The use of human tissues and the experiments performed in this study were approved by the Institutional Review Board of Chang Gung Memorial Hospital; the methods and experiments were carried out in accordance with the approved guidelines. Written informed consent was obtained from the patients. Control healthy kidney tissues were obtained from biopsies of trauma and hematoma patients with no history of kidney disease (n = 2); kidney tissues were also obtained from renal cancer patients (n = 20) and from end-stage kidney disease patients (n = 2) whose kidneys were removed or biopsied during transplant surgery (Table 1). For renal cancer tissues, the non-cancerous part of the tissue was dissected and analyzed in the present study.

### Histological analysis

Kidney tissues were mounted on paraffin blocks. The blocks were sectioned and tissue slices were heated on a hot plate at 70 °C for 30 min to remove paraffin. The sections were immersed into a fresh solution of xylene three times for 15 min to completely remove the remaining paraffin. The sections were rehydrated with 95%, 80%, and 70% ethanol for 5 min each time. Rehydrated specimens were washed with double-distilled water (ddH_2_O) for 5 min. Cell nuclei were stained with hematoxylin for 8 min. The dye was removed from the specimens with warm water for 10 min. Kidney specimens were rinsed in ddH_2_O and were dipped 10 times in 95% ethanol. The ethanol-treated specimens were counter-stained with eosin Y for 1 min. Specimens were dehydrated with 95% and 100% ethanol for 10 min each time, followed by a dehydration step in xylene for 10 min. The final dehydrated kidney specimens were mounted on glass slides with 50% glycerol and observed under a light microscope equipped with a digital camera.

De-paraffinized and rehydrated specimens were prepared as described for H&E staining. Rehydrated sections were stained with silver nitrate (5%), followed by exposure to UV light for 20 min. The solution was washed with ddH_2_O for 15 min. The sections were stained with sodium thiosulfate (5%) for 5 min, followed by washing with ddH_2_O for 15 min. The sections were stained with nuclear fast red (Sigma-Aldrich, St. Louis, MI) for 5 min. Stained specimens were dehydrated successively with 95% and 100% ethanol for 10 min each, prior to dehydration in xylene for 10 min. Kidney specimens were mounted onto glass slides and examined as described above.

### Electron microscopy and EDX analysis

Kidney samples were cut into small pieces less than 1 mm thick using a LGPS dissection microscope (Olympus, Tokyo, Japan). The tissues were fixed with 2.5% glutaraldehyde and 1% paraformaldehyde in 0.1 M cacodylate buffer at 4 °C overnight. Fixed samples were washed three times with 0.1 M cacodylate buffer in 8% sucrose (pH 7.2) on ice for 10 min. Washed tissues were incubated in phosphate-buffered saline (PBS; 137 mM NaCl, 2.7 mM KCl, 10 mM Na_2_HPO_4_) containing 1% osmium tetroxide and 1.5% potassium ferrocyanide for 2 h on ice. The tissues were washed with ddH_2_O on ice for 10 min three times. Washed tissues were stained on ice with 1% uranyl acetate in ddH_2_O for 1 h, prior to washing three times with ddH_2_O on ice. Kidney tissues were dehydrated with 30, 50, 70, 80, and 90% ethanol for 10 min each time, except when ethanol reached 70%, in which case the samples were stored at 4 °C overnight. Tissues were stained with 1% phosphotungstic acid in 95% ethanol for 15 min, prior to dehydration with 95% ethanol for 5 min. The specimens were immersed into propylene oxide at 40, 57, 67, and 100% in ethanol for 10 min each time, followed by another immersion in 100% propylene oxide for 5 min. Kidney tissues were infiltrated with 50, 70, and 100% Eponate 812 (Ted Pella, Redding, CA) for 1 h each time. Eponate 812-embedded kidney samples were prepared by incubating twice into 100% Eponate. Embedded samples were incubated into an oven at 60 °C overnight to allow resin polymerization.

Washed HS-NP pellets obtained as above were fixed with glutaraldehyde (2.5%) and paraformaldehyde (1%) in ddH_2_O for 4 h at 4 °C. Fixed pellets were washed with ddH_2_O three times for 10 min each time. Pellets were dehydrated with successive incubations into 30, 50, 70, 80, 90, 95, and 100% ethanol, for 10 min each time, except when ethanol reached 70%, in which the pellets were stored at 4 °C overnight. Other ethanol solutions were only put in contact with the pellets for 10 min. Dehydrated pellets were infiltrated with LR white embedding medium (Electron Microscopy Sciences, Hatfield, PA) using different ratios of ethanol and LR medium (3:1, 1:1, and 1:3) for 30 min each. The pellets were infiltrated with fresh LR medium (100%) overnight before polymerization. Pellets infiltrated with LR medium were incubated in an oven at 60 °C for 2 days to allow resin polymerization. Blocks of kidney and HS-NPs were sectioned to produce slices with a thickness of 70–100 nm using a Reichert Ultracut S microtome (Leica, Wetzlar, Germany). Tissue sections were stained with 4% uranyl acetate before visualization under a JEM 1230 transmission electron microscope (JEOL, Tokyo, Japan) operated at 100 kV. Electron diffraction patterns were obtained using the same system. Nickel grids were used as a support.

For EDX analysis, thin sections of kidney tissues were stained with uranyl acetate and washed with ddH_2_O as above, followed by drying in an electronic dessicator cabinet. Specimens were observed under a high-resolution JEM 2100 transmission electron microscope (JEOL) operated at 120 kV. EDX spectra were obtained in triplicate using an INCA Energy EDS system (Oxford Instruments, Abingdon, UK). Thin sections of HS-NPs were observed without staining.

### Preparation of mineralo-organic NPs

All starting solutions were adjusted to pH 7.4 and sterilized by filtration through 0.2 μm membranes prior to use. HS was obtained from healthy human volunteers using a conventional venipuncture technique. The use of human biological fluids in this study was approved by the Institutional Review Board of Linko Chang Gung Memorial Hospital and written informed consent was obtained from the volunteers. HS-NPs were prepared by adding 3 mM CaCl_2_ and Na_2_HPO_4_ each into DMEM (Gibco, Carlsbad, CA) containing 10% HS, followed by incubation for one week in cell culture conditions (37 °C, 5% CO_2_, humidified air). The particles were pelleted by centrifugation at 16,000 × *g* for 15 min at 4 °C and washed twice with HEPES buffer (20 mM HEPES, 1 mM CaCl_2_, 2 mM Na_2_HPO_4_, 150 mM NaCl) using the same centrifugation procedure.

### SDS-PAGE and Western blotting

SDS-PAGE and Western blot analysis were performed essentially as before^15^. Briefly, 0.2 μg (Fig. 5A,D) or 1.2 μg (Fig. 5B,C) of HS proteins, 47 μg (Fig. 5A,B,D) or 590 μg of HS-NP proteins (Fig. 5C), 0.1 μg of HSA (Fig. 5A–D), and 0.6 μg (Fig. 5A,C,D) or 0.15 μg of HSF (Fig. 5B) were dissolved in 5× “loading buffer” (0.313 M Tris-HCl pH 6.8, 10% SDS, 0.05% bromophenol blue, 50% glycerol, 12.5% β-mercaptoethanol) to a final concentration of 1×, prior to heating at 95 °C for 5 min and separation under denaturing and reducing conditions on a 10% SDS-PAGE using a mini-gel system (Hoefer, Holliston, MA). The NP control (used in lane 1 of Fig. 5A–D) consisted of mineral NPs prepared by adding 3 mM CaCl_2_ and Na_2_HPO_4_ each into DMEM (final volume of 1 ml), followed by incubation for one day in cell culture conditions; the particles were pelleted by centrifugation at 16,000 × *g* for 15 min, washed twice with HEPES buffer, and resuspended in 50 μl of HEPES buffer. An aliquot of 20 μl of resuspended particles was processed for SDS-PAGE as above. PVDF membranes were blocked for 1 h in 5% (w/v) defatted milk at room temperature. The primary antibodies generated in-house as described before^25^ were used at a dilution of 1:1,000 (α-apo-A1 and α-HS-NP), 1:3,000 (α-HSF), or 1:6,000 (α-HSA). The goat anti-rabbit horseradish peroxidase-conjugated secondary antibody was used based on the instructions provided by the manufacturer (Millipore, Billerica, MA). The blots were revealed using enhanced cheluminescence (Amersham Biosciences, Amersham, UK) and autoradiographic films.

### Immunogold labeling

Samples were prepared as for TEM observation. HS-NP blocks were sectioned into slices less than 70 nm thick. Sample sections on grids were blocked with 1% fish gelatin (Sigma) in 0.1 M HEPES buffer (pH 8.0) for 25 min. The grids were incubated with the following primary antibodies: α-HSA, 1:30; α-HSF, 1:50; α-apo-A1, 1:30; α-HS-NP, 1:60. The negative control contained no primary antibody (1% fish gelatin in HEPES buffer). The incubated sections were rinsed with HEPES buffer for 15 min. Rinsed sections were blocked with 1% fish gelatin in HEPES buffer for 20 min. The specimens were treated with the secondary 5-nm-gold-conjugate goat anti-rabbit IgG for 1 h. The specimens were washed with HEPES buffer for 10 min, prior to washing with ddH_2_O for 10 min. TEM observations were performed as above.

### Fluorescence microscopy

Histological kidney tissue slides prepared as above were blocked with 1% bovine serum albumin for 1 h at room temperature. The slides were incubated with primary polyclonal antibodies at a dilution of 1:4,000. After washing steps, the samples were incubated with the secondary antibodies goat anti-rabbit-FITC (492/520 nm) and goat anti-rabbit-TRITC (550/570 nm) (JacksonImmuno Research, West Grove, PA) at 1:100 for 1 h. The complexes were washed with PBST for 15 min. The fluorescent stain 4′,6-diamidino-2-phenylindole (DAPI) was used at 10 μg/ml for 15 min. DAPI-stained specimens were dehydrated with 95 and 100% ethanol for 10 min each, followed by dehydration in xylene for another 10 min. Dehydrated kidney specimens were mounted with Vectashield fluorescence H-1000 mounting medium (Vector Laboratories, Burlingame, CA) and were observed under a confocal microscope (LSM510 Meta; Zeiss, Oberkochen, Germany) equipped with a Spot Flex camera.

## Additional Information

**How to cite this article**: Wong, T.-Y. *et al.* Detection and characterization of mineralo-organic nanoparticles in human kidneys. *Sci. Rep.* **5**, 15272; doi: 10.1038/srep15272 (2015).

## Figures and Tables

**Table 1:** Information on human kidney tissue donor and ectopic calcification status.

#	Age	Gender	BUN (mg/dl) [6–21]	Creatinine (mg/dl) [M<1.27; F<1.03]	Albumin (g/dl) [3.5–5.5]	Total Cholesterol (mg/dl) [<200]	Urine Protein (mg/day) [50–80]	Urine Albumin (mg/dl)	Clinical Diagnosis	von Kossa Staining and NP Detection
1	25	F	–	–	–	–	–	–	Trauma	Negative
2	39	F	–	0.66	4.1	–	–	–	Hematoma	Negative
3	74	F	31	5.78	–	–	–	–	ESKD	Positive
4	56	M	–	–	–	–	–	–	ESKD	Positive
5	77	M	26	1.75	4.2	–	–	–	TCC	Positive
6	79	M	–	2.50	–	–	–	–	TCC	Negative
7	65	M	103	10.52	–	–	–	3+ (300)	RCC (T1b)	Positive
8	85	M	19	0.95	–	227	–	–	RCC (T1b)	Positive
9	66	F	–	1.10	–	–	–	–	TCC (T1a)	Positive
10	79	F	19	1.20	4.4	–	–	1+ (30)	TCC (T1a)	Negative
11	69	M	19	1.94	3.6	–	–	–	TCC (T1)	Positive
12	74	M	–	1.30	–	–	–	–	TCC (T3)	Negative
13	76	F	70	7.68	4.6	208	117	25	TCC (T1a)	Positive
14	63	M	16	2.52	–	–	–	–	TCC (T3)	Positive
15	61	F	–	1.54	–	–	–	–	TCC (T1a)	Negative
16	66	F	15	1.04	–	–	–	–	TCC (T3)	Positive
17	62	F	53	8.50	4.0	–	–	150	TCC (T2)	Positive
18	79	F	8	0.90	2.7	–	–	150	TCC	Positive
19	67	F	23	1.52	3.7	–	–	–	TCC	Positive
20	79	F	38	4.02	–	–	–	150	TCC (T1)	Positive
21	86	M	–	–	–	–	–	–	TCC	Positive
22	72	F	20	1.53	–	–	–	–	TCC	Positive

The range of normal renal test values is given within brackets. Renal cancer staging is given within parentheses in the “Clinical Diagnosis” column. von Kossa staining was considered positive when black precipitates were detected by optical microscopy; NP detection was confirmed using TEM. Biopsies from hematoma and trauma patients with no history or sign of kidney function abnormalities were used as healthy controls. Staging of renal cancer from stages 1 to 4 (T1–T4) is given within parentheses. The dash symbol (–) denotes unavailable information. Abbreviations: BUN: blood urea nitrogen; M: male; F: female; NP: nanoparticle; ESKD: end-stage kidney disease; TCC: transitional cell carcinoma; RCC: renal cell carcinoma.
